# Corticospinal Reorganization after Locomotor Training in a Person with Motor Incomplete Paraplegia

**DOI:** 10.1155/2013/516427

**Published:** 2012-12-26

**Authors:** Nupur Hajela, Chaithanya K. Mummidisetty, Andrew C. Smith, Maria Knikou

**Affiliations:** ^1^Electrophysiological Analysis of Gait and Posture Laboratory, Sensory Motor Performance Program, Rehabilitation Institute of Chicago, 345 East Superior Street, Chicago, IL 60611, USA; ^2^Department of Physical Medicine and Rehabilitation, Northwestern University Feinberg School of Medicine, Chicago, IL 60611, USA; ^3^Department of Physical Therapy and the Graduate Center, The City University of New York, Staten Island, NY 10314, USA

## Abstract

Activity-dependent plasticity as a result of reorganization of neural circuits is a fundamental characteristic of the central nervous system that occurs simultaneously in multiple sites. In this study, we established the effects of subthreshold transcranial magnetic stimulation (TMS) over the primary motor cortex region on the tibialis anterior (TA) long-latency flexion reflex. Neurophysiological tests were conducted before and after robotic gait training in one person with a motor incomplete spinal cord injury (SCI) while at rest and during robotic-assisted stepping. The TA flexion reflex was evoked following nonnociceptive sural nerve stimulation and was conditioned by TMS at 0.9 TA motor evoked potential resting threshold at conditioning-test intervals that ranged from 70 to 130 ms. Subthreshold TMS induced a significant facilitation on the TA flexion reflex before training, which was reversed to depression after training with the subject seated at rest. During stepping, corticospinal facilitation of the flexion reflex at early and midstance phases before training was replaced with depression at early and midswing followed by facilitation at late swing after training. These results constitute the first neurophysiologic evidence that locomotor training reorganizes the cortical control of spinal interneuronal circuits that generate patterned motor activity, modifying spinal reflex function, in the chronic lesioned human spinal cord.

## 1. Introduction

A plethora of studies have shown that the isolated mammalian spinal cord can generate muscle activation patterns suited for locomotion in absence of inputs from the brain [1, 2]. This work led to the notion that neural drive from the brain is needed mostly when environmental constraints increase such as stepping over an obstacle or on an uneven surface [3–5]. However, corticospinal neurons are active during simple locomotion and exhibit a profound step-related modulation in the cat [6–8]. Similarly, corticospinal pathways to leg muscles are activated in a phase-dependent manner during simple treadmill walking in humans, long-latency reflexes of the tibialis anterior (TA) muscle are partly mediated by a transcortical pathway, and impaired transmission in the corticospinal tract is related to gait disability of individuals with a spinal cord injury (SCI) [9–11]. These findings support the notion of a substantial cortical involvement in human walking. 

Because of motor incomplete SCI, the spinal cord is not completely severed and thus some descending fiber tracts and segmental spinal cord circuits remain intact; it is logical to hypothesize that cortical control of spinal neural circuits is reorganized after locomotor training. This hypothesis is supported by the fact that activity-dependent neuroplasticity takes place simultaneously in multiple sites of the central nervous system due to training [12, 13]. Improvements in walking ability have been achieved with locomotor training post-SCI, and changes have been reported in walking speed, step length, and step symmetry [14]. The reported changes are likely the result of task-specific sensorimotor feedback that reorganizes corticospinal and spinal pathways in a functional manner [15, 16]. For example, in 4 people with SCI, functional magnetic resonance imaging showed a greater activation in sensorimotor cortical and cerebellar regions after 36 sessions of body weight supported (BWS) robotic gait training [17]. In individuals with incomplete SCI, 3 to 5 months of daily locomotor training increased the size of the motor evoked potentials (MEPs) in 9 out of 13 muscles tested, increased the maximal MEP, and changed the slope of the MEP input-output curve [18]. The changes in MEP size were significantly correlated to the degree of locomotor recovery, suggesting that corticospinal plasticity was involved, at least in part, in the recovery of walking ability after training [18]. 

Collectively, we hypothesized that locomotor training reorganizes the cortical control of spinal interneuronal pathways that generate patterned motor activity during locomotion. We tested our hypothesis by establishing the effects of subthreshold transcranial magnetic stimulation (TMS) over the primary motor cortex region on the spinal polysynaptic flexion reflex before and after BWS robotic gait training in one person with motor incomplete paraplegia while at rest and during robotic-assisted stepping. We selected this reflex because the interneuronal circuits that generate the flexion reflex also participate in pattern generation during locomotion, and this reflex is susceptible to descending control [19].

## 2. Materials and Methods

### 2.1. Subject

A 52-year-old woman, 11-year post-SCI, at the level of thoracic 7 due to fall, participated in this study following written consent to the experimental procedures approved by the Northwestern University (Chicago, IL, USA) Institutional Review Board committee and conducted in accordance with the Declaration of Helsinki. Based on neurological examination according to the American Spinal Injury Association guidelines, the subject had an AIS grade D impairment scale at the time of admission to the study. The subject received 35 training sessions (1 hour/day, 5 days/week) with a robotic exoskeleton (Lokomat, Hocoma, Switzerland). Before and after training, electromyographic (EMG) activity was recorded from medial gastrocnemius (MG), peroneus longus (PL), gracilis (GRC) and medial hamstrings (MH) of the right leg, and tibialis anterior (TA) and soleus (SOL) from both legs with bipolar differential electrodes of fixed interelectrode distance (Motion Lab Systems, Baton Rouge, LA, USA). EMG and foot switches data were collected at 2000 Hz with custom-written acquisition software (Labview, National Instruments, Austin, TX, USA). Results of clinical evaluation tests and treadmill parameters before and after training are summarized in Table 1.

### 2.2. Neurophysiological Tests Conducted before and after Training

With the subject seated at rest, the sural nerve of the left leg was stimulated with a pulse train of 30 ms duration once every 10 s with a constant current stimulator (DS7A, Digitimer, Hertfordshire, UK) [20, 21]. Stimulation was delivered by two disposable pregelled Ag-AgCl electrodes (Conmed Corporation, NY, USA) placed on the lateral malleolus and maintained in place via an athletic wrap. Reflex responses were recorded from the ipsilateral TA muscle. Sural nerve stimulation during testing was delivered at 1.3 times the reflex threshold. No limb movement or pain was present upon stimulation.

Single TMS pulses over the right primary motor cortex (M1) were delivered with a Magstim 200 stimulator (Magstim, Whitland, UK). The double-coned coil was oriented on the skull to produce an induced current in the posterior-to-anterior direction. The optimal position for TMS was determined by varying the position of the coil from the vertex with gradually increasing intensities, until an MEP in the contralateral (left) TA muscle was observed at the lowest stimulation intensities with the subject seated at rest. MEP resting threshold was defined as the stimulus intensity at which three MEPs of at least 100 *μ*V of peak-to-peak amplitude were evoked following five consecutive stimuli with the subject at rest.

After cortical and sural nerve stimulation sites were established, the effects of TMS delivered at 0.9 TA MEP resting threshold on the TA flexion reflex at the conditioning-test (C-T) intervals of 70, 90, 110, and 130 ms were determined with the seated subject. Ten flexion reflexes, each evoked once every 10 s, were recorded under control conditions and following subthreshold TMS. Then, the subject was transferred to standing at 50% BWS, and the TA flexion reflex and MEP thresholds were reestablished. During robotic-assisted stepping, the flexion reflex was conditioned by TMS at 0.9 × TA MEP resting threshold at the C-T intervals of 70 ms and 110 ms before and after training. The subject stepped at 50% BWS and at 1.8 Km/h treadmill speed for both data collection sessions. Stimulation was triggered every 3 steps, based on the signal from the left-foot switch, which was sent randomly across different phases of a step cycle that was divided into 16 equal time windows or bins [21, 22].

### 2.3. Data Analysis

EMG signals during BWS-assisted stepping from the steps before sural nerve and transcranial magnetic stimulation were full-wave rectified, high-pass filtered at 20 Hz, and low-pass filtered at 500 Hz. After full-wave rectification, linear envelopes were obtained at 20 Hz low-pass filter, and the mean EMG amplitude across all steps was determined. Integrated EMG was defined as the area under the linear envelope. This analysis was conducted separately for each muscle during BWS-assisted stepping for both sessions. The overall average of the EMG linear envelope (including all bins) from each muscle was also estimated and compared before and after training with a paired *t*-test.

Flexion reflexes were measured as the area under the full-wave rectified EMG response. The conditioned TA flexion reflex (*n* = 10) recorded at each C-T interval before and after training with the seated subject was expressed as a percentage of the mean size of the associated control flexion reflex. Statistically significant differences between the conditioned flexion reflexes recorded at different C-T intervals before and after training were established with a multiple ANOVA at 2 × 4 levels (2: pre-/post-training, 4: C-T intervals) along with Holm-Sidak tests for repeated measures. At each bin of the step cycle, the full-wave rectified area of the TA flexion reflex response was calculated and averaged separately for steps with and without sural nerve stimulation and TMS [22]. The average of TA EMGs of non-stimulated steps was subtracted from the average of EMGs of stimulated steps (conditioned reflex) at identical time windows for each bin and was expressed as a percentage of the control flexion reflex recorded with the seated subject. Statistically significant differences between the conditioned flexion reflexes recorded at each bin of the step cycle before and after training were established with a two-way ANOVA at 2 × 16 levels (2: pre-/post- training, 16: bins of the step cycle) along with Holm-Sidak tests for repeated measures. This analysis was conducted separately for flexion reflexes at the C-T intervals of 70 and 110 ms. Alpha was set at 95% for all statistical tests.

## 3. Results

**Figure 1:**
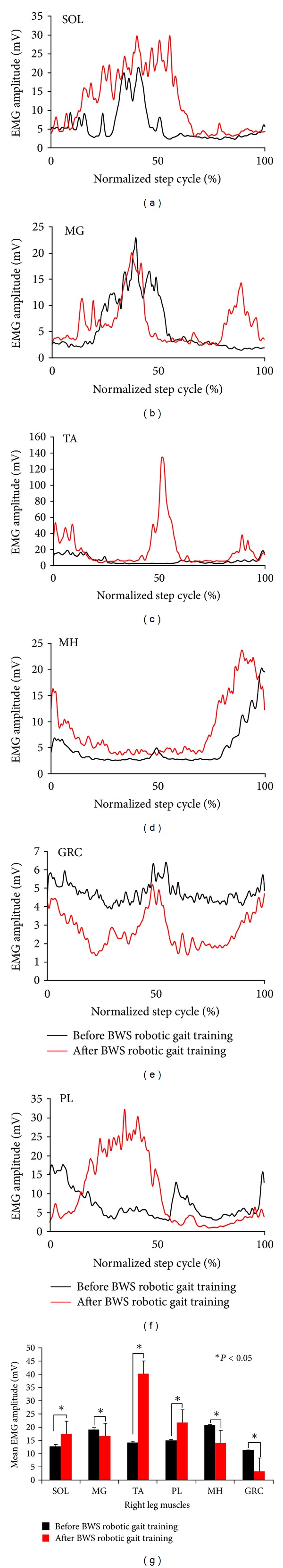
EMG activity during robotic-assisted stepping before and after training. (a)–(f) EMG activity of the right side muscles during robotic-assisted stepping at 50% BWS and at 1.8 Km/h before and after training as a function of the step cycle. (g) Mean EMG amplitude for stepping before (black squares) and after (red squares) 35 sessions of robotic gait training. EMG: electromyography; SOL: soleus; MG: medial gastrocnemius; TA: tibialis anterior; PL: peroneus longus; MH: medial hamstrings; GRC: gracilis.

The latency of the TA flexion reflex following sural nerve stimulation measured from the onset of the pulse train was 160 ms, while the latency of the TA MEP was 40 ms before and after training. The EMG activation patterns as a function of the step cycle changed significantly after robotic gait training. Specifically, the SOL EMG burst duration was prolonged during the stance phase (Figure 1(a)), MG displayed an EMG burst during the stance and late swing phases (Figure 1(b)); while the PL EMG burst was enhanced throughout the stance phase (Figure 1(f)). The EMG activation profiles of SOL, MG, PL, and MH muscles are similar to those observed in control subjects during robotic-assisted stepping, but an absent TA activity is noted at early stance and late swing phases when compared to the TA EMG profile observed commonly in control subjects (see Figure 1(b) in [23]). The most pronounced change noted is in the TA muscle in which a burst of activity was present at late stance phase (Figure 1(c)), while before training a clear TA EMG activity was absent. An increase in the overall EMGs amplitude computed across all bins of the step cycle was noted in all leg muscles (*P* < 0.05; Figure 1(g)).

**Figure 2:**
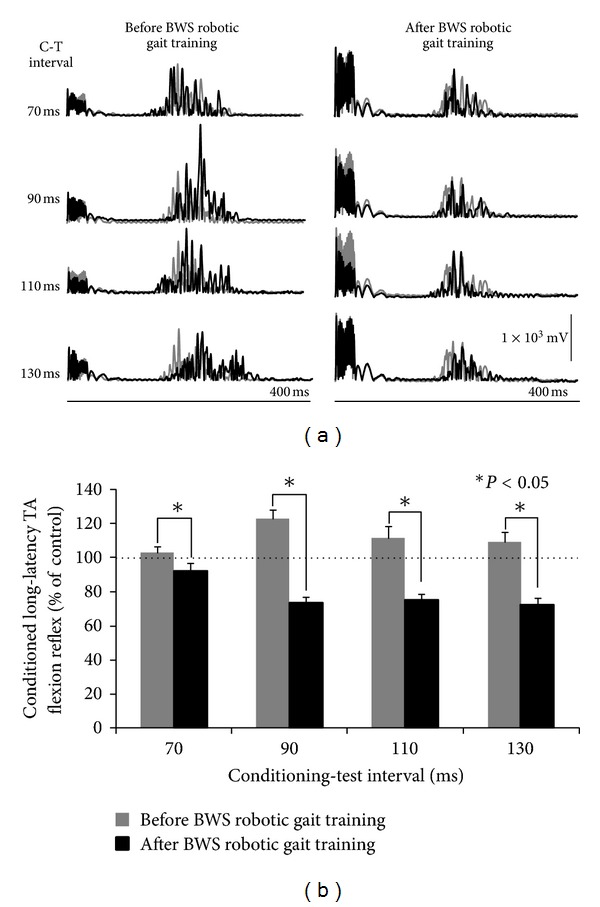
Effects of subthreshold TMS on the TA flexion reflex while seated before and after BWS robotic gait training. (a) Full-wave rectified waveform averages (*n* = 10) of the control tibialis anterior (TA) flexion reflex (grey line) and the conditioned flexion reflex following single pulse transcranial magnetic stimulation (TMS) of the right primary motor cortex at 0.9 TA motor evoked potentials (MEPs) resting threshold. (b) Mean amplitude of the conditioned TA flexion reflexes recorded before and after BWS robotic gait training with the seated subject. The conditioning-test interval is denoted on the abscissa. Asterisks indicate statistically significant differences between the conditioned TA flexion reflexes recorded before and after training. Error bars denote the SEM.

In Figure 2(a), full-wave rectified waveform averages of the TA flexion reflex recorded under control conditions (grey line) and following TMS at 0.9 × MEP resting threshold (black lines) are indicated for recordings taken before and after training. In Figure 2(b), the amplitude of the conditioned TA flexion reflex as a percentage of the control flexion reflex before and after training is indicated. A MANOVA showed that the conditioned long-latency TA flexion reflex was statistically significantly different before and after training (*F*_1,8_ = 81.7, *P* < 0.05), and that the amplitude of the conditioned flexion reflex did not vary across C-T intervals tested for recordings taken before and after training (*F*_3,24_ = 1.4, *P* > 0.05).

**Figure 3:**
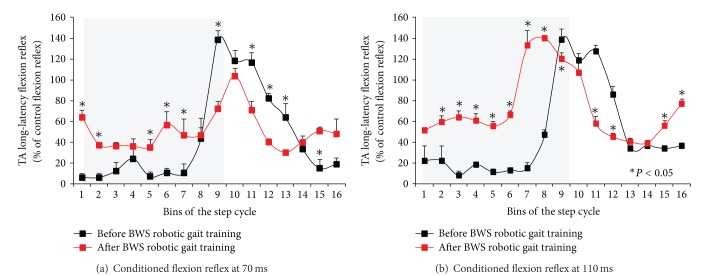
Changes in cortical control of the flexion reflex after 30 sessions of BWS robotic gait training during robotic-assisted stepping. The mean normalized long-latency tibialis anterior (TA) flexion reflex following single pulse transcranial magnetic stimulation (TMS) of the right primary motor cortex at 0.9 × TA motor evoked potentials (MEPs) at the conditioning-test interval of 70 (a) and 110 (b) ms is indicated as a function of the step cycle. Asterisks indicate suppressive and/or facilitatory conditioned flexion reflexes after locomotor training compared to those observed before training based on the *P* value computed from pairwise multiple comparisons (two-way ANOVA along with Holm-Sidak tests). Grey squares denote the stance phase. Error bars denote the SEM.

The changes observed after training during robotic-assisted stepping were more complex compared to the uniform flexion reflex depression observed with the seated subject. In Figure 3, the mean amplitude of the long-latency TA flexion reflex following TMS at 0.9 × MEP resting threshold at the C-T intervals of 70 ms and 110 ms as a function of the step cycle is indicated. A two-way ANOVA at 2 × 16 levels (2: pre/post training, 16: bins of the step cycle) showed that the TA flexion reflex at the C-T interval of 70 ms was statistically significantly different across bins (*P* < 0.001). Pairwise multiple comparisons (Holm-Sidak tests) showed that the conditioned flexion reflex at bins 1, 2, 5, 6, 7, 9, 11, 12, 13, 15, and 16 was statistically significantly different before and after training (*P* < 0.05). These results suggest that after training, the conditioned TA flexion reflex at the C-T interval of 70 ms was significantly enhanced during the stance phase, followed by a depression from early swing until midswing (bins 9–13) when compared to the conditioned flexion reflex recorded before training (Figure 3(a)). A two-way ANOVA at 2 × 16 levels (2: pre-/post- training, 16: bins of the step cycle) showed that the TA flexion reflex at the C-T interval of 110 ms was statistically significantly different across bins (*P* < 0.001). Pairwise Holm-Sidak tests for multiple comparisons showed that the conditioned flexion reflex throughout the stance phase (bins 2–8) was facilitated, followed by a significant depression at early swing phase (bins 11, 12) and a significant facilitation at swing-to-stance transition phase (bins 15, 16) (*P* < 0.05) (Figure 3(b)).

## 4. Discussion

Locomotor training with a robotic exoskeleton reorganized the cortical control of spinal interneuronal circuits and modified the flexion reflex function at rest and during assisted stepping in a person with a chronic motor incomplete SCI. Before training and with the seated subject, subthreshold TMS resulted in facilitation of the long-latency TA flexion reflex, but after training a pronounced reflex depression was evident. Corticospinal actions on the flexion reflex changed in a more complex pattern during robotic-assisted stepping. After training, corticospinal facilitation of the flexion reflex at early and midstance was replaced with depression at early and midswing followed by facilitation at late swing. Two possible explanations for these changes are that the residual intact supraspinal connections were reorganized or that new supraspinal connections with spinal networks were formed with locomotor training as a result of activity-dependent mechanisms driven by task-specific sensory cues [12, 13, 24]. These sensory cues included load alternation and leg positioning with kinematics of the hips, knees, and ankles timed to the step cycle in a physiologic pattern predetermined by the robotic exoskeleton system.

Activity-dependent plasticity involves both physiological and structural changes that alter the anatomical connectivity of neurons [24–26]. We are not able to effectively assess which anatomical connections exist after the injury and which change with training. Nonetheless, the neuronal pathways and circuits that may have changed due to training are intracortical and interhemispheric inhibitory circuits, corticospinal monosynaptic connections with TA alpha motoneurons, and oligo- or polysynaptic cortical connections with flexion reflex afferent (FRA) interneurons. The rationale for proposing these neuronal pathways is based on the demonstrated effects of subthreshold TMS on the spinal motoneurons through intracortical and interhemispheric inhibitory circuits [27–30], and on the fact that TMS delivered 0.9 × MEP resting threshold, it may have produced corticospinal motor volleys that affected the excitability state of FRA interneurons and TA alpha motoneurons. Because of the long latency of the flexion reflex as well as that the conditioning reflex effects were observed at long C-T intervals, it is likely that monosynaptic excitation of TA alpha motoneurons by corticospinal volleys was absent and that corticospinal descending volleys affected FRA interneurons after a polysynaptic relay [30].

Sural nerve stimulation largely excited A*β* (or group II) sensory afferents mediating tactile information. The conduction velocity of these afferents ranges from 30 to 70 m s^−1^ while during contraction is 45 m s^−1^ [31]. Further, the conduction velocity of the early D (or direct) wave after scalp stimulation recorded with epidural electrodes at the thoracic 5 ranges from 62 to 70 m s^−1^ [32]. This means that impulses from A*β* fibers reached the spinal cord about 14–30 ms after the first pulse of the reflex stimulus pulse train, while corticospinal motor volleys reached the spinal cord approximately 10 ms following TMS. Because changes in motoneuronal excitation following M1 excitation can last as long as 80 to 100 ms, it is apparent that at the C-T intervals used in this study, there was ample time for TMS to affect the excitability state of FRA interneurons that produce polysynaptic reflex actions on *α*-motoneurons.

Our finding—that corticospinal control of spinal cord neural circuits was reorganized after locomotor training—is important and constitutes the first proof of principle for this therapeutic strategy based on neurophysiological evidence. The changes in the corticospinal pathways we observed here may be linked to improvements of walking ability and balance. After locomotor training, the person was able to walk 335 m within 6 min compared to 269 m before training, while significant improvements were noted on balance-related motor tasks and speed of walking (Table 1). Clinical studies have demonstrated that locomotor training improves walking ability and cardiovascular function in people with motor incomplete SCI [33, 34]. Taken together, we propose that recovery of walking ability is mediated through reorganization of corticospinal actions on spinal interneuronal circuits modifying reflex function during walking.

At this point, it should be noted that a key limitation of this study is that data was collected from one patient, and thus generalization to a specific SCI population should be cautioned. Further, the subject received only 35 sessions of robotic gait training. Rehabilitation of these patients to achieve restoration of movement and walking is a long-term process, while reorganization of corticospinal control of spinal reflex circuits may differ after 60 or 90 training sessions. Thus, the corticospinal reorganization we observed here, evident by the modulation pattern of the flexion reflex following TMS with the seated subject and during robotic-assisted stepping, may reflect a specific stage of the task-dependent plasticity of corticospinal neural circuits [35]. It is apparent that further research is needed to outline the neurophysiological changes associated with corticospinal reorganization due to locomotor training and the role of corticospinal neural plasticity in restoration of walking ability after SCI.

## 5. Conclusion

We demonstrate in this study, for the first time, that cortical actions on spinal interneuronal circuits are reorganized after locomotor training in one person with chronic motor incomplete SCI. This neural reorganization may be the result of newly formed supraspinal connections with spinal networks or potentiation of inactive residual intact supraspinal connections due to training. Further research is needed to link reorganization of corticospinal neural pathways to locomotor training-mediated restoration of walking ability as well as phases of neuroplasticity over time.

## Figures and Tables

**Table 1:** Treadmill walking parameters and functional outcomes^1^.

BWS (%)	Speed (Km/h)	R and L foot lifters	Guidance force by the Robot (%)	Clonus	Extensor spasticity (SCATS)	Manual muscle testing	6 min walk	30 sec chair-stand test	Time up and go
				Before robotic gait training					

50	1.8	None	100	1L/0R	0L/0R	R leg = 24/25 L leg = 16/25	269 m using quad cane	12 reps	11.3 sec using quad can

				After robotic gait training					

15	3.2	None	15	1L/0R	0L/0R	R leg = 24/25 L leg = 17/25	335 m using quad cane	21 reps	9.1 sec using quad cane

^1^BWS: body weight support; extensor spasticity grade is based on the spinal cord assessment tool for spasticity (SCATS): where subjects are positioned supine, the lower limb is rapidly moved into passive extension, and the severity of quadriceps contraction is scored; R: right, L: left; 0: no reaction to stimulus; 1: mild quadriceps contraction between 1–3 seconds.

## Abbreviations

BWS: Body weight support
C-T: Conditioning-test
EMG: Electromyographic
MEP: Motor evoked potentials
SCI: Spinal cord injury
TA: Tibialis anterior
TMS: Transcranial magnetic stimulation.

## References

[1] Rossignol S, Barrière G, Frigon A (2008). Plasticity of locomotor sensorimotor interactions after peripheral and/or spinal lesions. *Brain Research Reviews*.

[2] Edgerton VR, Tillakaratne NJK, Bigbee AJ, de Leon RD, Roy RR (2004). Plasticity of the spinal neural circuitry after injury. *Annual Review of Neuroscience*.

[3] Beloozerova IN, Sirota MG (1993). The role of the motor cortex in the control of accuracy of locomotor movements in the cat. *Journal of Physiology*.

[4] Beloozerova IN, Farrell BJ, Sirota MG, Prilutsky BI (2010). Differences in movement mechanics, electromyographic, and motor cortex activity between accurate and nonaccurate stepping. *Journal of Neurophysiology*.

[5] Drew T, Andujar JE, Lajoie K, Yakovenko S (2008). Cortical mechanisms involved in visuomotor coordination during precision walking. *Brain Research Reviews*.

[6] Armstrong DM, Drew T (1984). Discharges of pyramidal tract and other motor cortical neurones during locomotion in the cat. *Journal of Physiology*.

[7] Armstrong DM, Drew T (1984). Locomotor-related neuronal discharges in cat motor cortex compared with peripheral receptive fields and evoked movements. *Journal of Physiology*.

[8] Drew T (1993). Motor cortical activity during voluntary gait modifications in the cat. I. Cells related to the forelimbs. *Journal of Neurophysiology*.

[9] Barthélemy D, Grey MJ, Nielsen JB, Bouyer L (2011). Involvement of the corticospinal tract in the control of human gait. *Progress in Brain Research*.

[10] Nielsen J, Petersen N, Fedirchuk B (1997). Evidence suggesting a transcortical pathway from cutaneous foot afferents to tibialis anterior motoneurones in man. *Journal of Physiology*.

[11] Barthélemy D, Willerslev-Olsen M, Lundell H (2010). Impaired transmission in the corticospinal tract and gait disability in spinal cord injured persons. *Journal of Neurophysiology*.

[12] Dobkin BH (2000). Functional rewiring of brain and spinal cord after injury: the three Rs of neural repair and neurological rehabilitation. *Current Opinion in Neurology*.

[13] Wolpaw JR, Tennissen AM (2001). Activity-dependent spinal cord plasticity in health and disease. *Annual Review of Neuroscience*.

[14] Dobkin B, Apple D, Barbeau H (2006). Weight-supported treadmill vs over-ground training for walking after acute incomplete SCI. *Neurology*.

[15] Knikou M (2010). Neural control of locomotion and training-induced plasticity after spinal and cerebral lesions. *Clinical Neurophysiology*.

[16] Knikou M (2012). Plasticity of corticospinal neural control after locomotor training in human spinal cord injury. *Neural Plasticity*.

[17] Winchester P, McColl R, Querry R (2005). Changes in supraspinal activation patterns following robotic locomotor therapy in motor-incomplete spinal cord injury. *Neurorehabilitation and Neural Repair*.

[18] Thomas SL, Gorassini MA (2005). Increases in corticospinal tract function by treadmill training after incomplete spinal cord injury. *Journal of Neurophysiology*.

[19] Sherrington CS (1910). Flexion-reflex of the limb, crossed extension-reflex and reflex stepping and standing. *The Journal of Physiology*.

[20] Knikou M (2007). Plantar cutaneous input modulates differently spinal reflexes in subjects with intact and injured spinal cord. *Spinal Cord*.

[21] Knikou M (2010). Plantar cutaneous afferents normalize the reflex modulation patterns during stepping in chronic human spinal cord injury. *Journal of Neurophysiology*.

[22] Knikou M, Angeli CA, Ferreira CK, Harkema SJ (2009). Flexion reflex modulation during stepping in human spinal cord injury. *Experimental Brain Research*.

[23] Knikou M, Hajela N, Mummidisetty CK, Xiao M, Smith AC (2011). Soleus H-reflex phase-dependent modulation is preserved during stepping within a robotic exoskeleton. *Clinical Neurophysiology*.

[24] Sanes JN, Donoghue JP (2000). Plasticity and primary motor cortex. *Annual Review of Neuroscience*.

[25] Feldman DE (2009). Synaptic mechanisms for plasticity in neocortex. *Annual Review of Neuroscience*.

[26] Butz M, Wörgötter F, van Ooyen A (2009). Activity-dependent structural plasticity. *Brain Research Reviews*.

[27] Valls-Solé J, Pascual-Leone A, Wassermann EM, Hallett M (1992). Human motor evoked responses to paired transcranial magnetic stimuli. *Electroencephalography and Clinical Neurophysiology*.

[28] Di Lazzaro V, Restuccia D, Oliviero A (1998). Magnetic transcranial stimulation at intensities below active motor threshold activates intracortical inhibitory circuits. *Experimental Brain Research*.

[29] Kujirai T, Caramia MD, Rothwell JC (1993). Corticocortical inhibition in human motor cortex. *Journal of Physiology*.

[30] Cowan JMA, Day BL, Marsden C, Rothwell JC (1986). The effect of percutaneous motor cortex stimulation on H reflexes in muscles of the arm and leg in intact man. *Journal of Physiology*.

[31] Rossi A, Zalaffi A, Decchi B (1996). Interaction of nociceptive and non-nociceptive cutaneous afferents from foot sole in common reflex pathways to tibialis anterior motoneurones in humans. *Brain Research*.

[32] Inghilleri M, Berardelli A, Cruccu G, Priori A, Manfredi M (1989). Corticospinal potentials after transcranial stimulation in humans. *Journal of Neurology Neurosurgery and Psychiatry*.

[33] Dobkin B, Barbeau H, Deforge D (2007). The evolution of walking-related outcomes over the first 12 weeks of rehabilitation for incomplete traumatic spinal cord injury: the multicenter randomized Spinal Cord Injury Locomotor trial. *Neurorehabilitation and Neural Repair*.

[34] Turiel M, Sitia S, Cicala S (2011). Robotic treadmill training improves cardiovascular function in spinal cord injury patients. *International Journal of Cardiology*.

[35] Wolpaw JR, O’Keefe JA (1984). Adaptive plasticity in the primate spinal stretch reflex: evidence for a two-phase process. *Journal of Neuroscience*.

